# The Attenuated Psychosis Syndrome and Facial Affect Processing in Adolescents With and Without Autism

**DOI:** 10.3389/fpsyt.2020.00759

**Published:** 2020-08-03

**Authors:** Arija Maat, Sebastian Therman, Hanna Swaab, Tim Ziermans

**Affiliations:** ^1^ Department of Psychiatry, UMC Utrecht, University of Utrecht, Utrecht, Netherlands; ^2^ Mental Health Unit, National Institute for Health and Welfare, Helsinki, Finland; ^3^ Clinical Child & Adolescent Studies, Leiden University, Leiden, Netherlands; ^4^ Department of Psychology, University of Amsterdam, Amsterdam, Netherlands

**Keywords:** autism spectrum disorder, psychosis, schizophrenia, ultra-high risk, social cognition, emotion perception, attenuated positive symptoms

## Abstract

**Background:**

Autism and schizophrenia spectrum disorders both represent severely disabling neurodevelopmental disorders with marked impairments in social functioning. Despite an increased incidence of psychosis in autism, and substantial overlap in symptoms and cognitive markers, it is unclear whether such phenotypes are specifically related to risk for psychosis or perhaps reflect more general, idiosyncratic autism traits. The attenuated psychosis syndrome (APS) is primarily defined by the presence of attenuated psychotic symptoms, which currently constitute the best and most-replicated clinical predictors of psychosis, and are common in clinical youth with and without autism. The aims of this study were to test the hypothesis that facial affect processing is impaired in adolescents with APS and to explore whether such deficits are more indicative of psychotic or autistic phenotypes on a categorical and dimensional level.

**Materials and Method:**

Fifty-three adolescents with APS and 81 typically developing controls (aged 12–18) were included. The APS group consisted of adolescents with (n = 21) and without (n = 32) a diagnosis of autism spectrum disorder. Facial affect recognition was assessed with the Amsterdam Neuropsychological Tasks using a cascade model of cognitive processing, in which disturbances in “lower-level” cognitive abilities (pattern recognition), affect “higher-level” cognitive processes (face recognition and facial affect recognition). For associations with schizotypal and autistic-like traits the Schizotypal Personality Questionnaire and Social Communication Questionnaire were used in a confirmatory item factor analysis framework.

**Results:**

Contrary to expectation, APS in adolescents was not associated with impairments in pattern, face, or facial affect recognition. However, the APS group with autism spectrum disorder showed a general latency in response time to social and non-social stimuli. Dimensionally assessed schizotypal and autistic-like traits did not predict the accuracy or the speed of face or facial affect recognition.

**Conclusion:**

Facial affect processing performance was not associated with APS in adolescence and represents an unlikely early vulnerability marker for psychosis. APS individuals with a more autistic-like profile were characterized by slower responses to social- and non-social stimuli, suggesting that the combined effect of APS and autism spectrum disorder on cognition is larger than for APS alone.

## Introduction

Autism spectrum disorder (ASD) and schizophrenia spectrum disorders (SSD) both represent severely disabling neurodevelopmental disorders with marked impairments in social functioning. ASD and SSD co-occur more often than would be expected by chance (1, 2), and have been found to share both phenotypic similarities as well as multiple risk factors (3). Recently, more parallels between ASD and SSD have come to light, such as overlapping genetic mechanisms and brain developmental trajectories (4–6). Furthermore, studies are increasingly focussing on dimensional rather than categorical approaches, with the aim of testing the hypothesis that both conditions represent extremes on an extended continuum of symptomatic severity. These efforts provide evidence for elevated rates of autistic traits in individuals diagnosed with SSD, and also report that these traits negatively affect clinical outcome such as quality of life and global functioning (3, 7–10)

Of interest for the present study is the striking overlap of many cognitive traits between ASD and SSD (11), especially within the domain of social cognition (8, 12–15). For example, investigations of emotion recognition in both ASD and SSD reveal consistent impairments compared to healthy controls (13, 16). However, in a recent direct comparison of substantial clinical samples, adults with ASD seemed significantly more impaired than adults with SSD in emotion perception from faces (15). Although this difference may become less pronounced with increasing age due to progressive cognitive deterioration in SSD (12), cross-sectional studies show that clear social cognitive impairments are already present at first onset of psychosis (17, 18). In addition, numerous studies have reported social cognitive deficits in individuals at risk for psychosis, that is, in both first-degree relatives of schizophrenia patients (19), as well as in individuals with a clinical or “ultra” high risk (UHR) for psychosis (20). However, it remains unclear as to how and when these impairments develop (21) and whether they convey a similar risk for psychosis in UHR and ASD.

The UHR criteria were developed to help identify young help-seeking individuals at imminent risk for developing a psychotic disorder (22). In the past decades it has been established that approximately 20% of UHR positive individuals will develop a psychotic disorder within two years of identification (23), depending on the study population base rate and type of inclusion criteria (24). Also, transition rates have been slowly declining over time (25) and tend to be somewhat lower in young adolescent UHR populations (26, 27), though they remain staggeringly high compared to the general population. Of the different UHR inclusion criteria, attenuated positive symptoms are by far the most commonly represented and currently constitute the best and most-replicated clinical predictors of psychosis (28). Together with the proposal to include an attenuated psychosis syndrome (APS), which is almost exclusively defined by the presence of attenuated positive symptoms, into the DSM (29), this has led to a partial shift in research focus toward (presumably) more homogenous APS samples to improve replicability of factors associated with risk for psychosis.

Like APS, a childhood diagnosis of ASD is also characterized by much greater odds to develop psychosis compared to the general population (2, 30). Psychotic symptoms are not included in diagnostic ASD criteria, yet many individuals diagnosed with ASD report psychotic symptoms, even at a young age (31–34). Given the elevated risk for psychosis in young people with APS and ASD, as well as the shared phenomenology, direct comparisons between the two are notably absent from the literature. As an exception, a recent longitudinal study reported that UHR patients with and without premorbid ASD showed similar APS at baseline and conversion rates to full-blown psychosis for both groups (35). However, baseline social cognitive performance (i.e. social perception and theory of mind) was more affected in UHR with ASD. This suggests that social cognitive deficits contain a different level of risk for transition to psychosis in UHR individuals with and without ASD.

As highlighted above, social cognition deficits are commonly proposed as a potential early vulnerability marker for psychosis. However, actual associations with transition to psychosis are few and inconsistent in high-risk research (36). Regardless, some studies suggest a positive predictive value of specific impairments in facial affect processing for the transition to psychosis (37–39) and negative outcomes (40), which warrants further investigation. Facial affect processing is associated with non-affective facial processing and, in addition to involvement of the limbic regions, partially requires activation of the same cortical structures, such as the fusiform face area (41). In turn, non-affective facial processing involves similar brain regions as non-affective visuospatial processing, i.e. the processing of patterns or objects, but evokes differential activity patterns (42). In this view, more complex and “higher-level” social cognitive skills such as facial affect processing and non-affective facial identity recognition may partially rely on “lower-level”, non-social visual processing skills for optimal functioning, which is often reported as being aberrant in schizophrenia (43–45). Simultaneous assessment of these three separate, yet interrelated, levels of visual processing can therefore further inform us on their associations with psychotic and/or autistic behavior.

The aims of this study were to test facial affect processing in help-seeking adolescents (12–18 years) suffering from APS with and without ASD and to explore whether any deficits are more indicative of psychotic or autistic phenotypes on a categorical and dimensional level. Furthermore, facial affect recognition was assessed within the context of a cascade model of cognitive processing, in which disturbances in “lower-level” cognitive abilities (pattern recognition), affect “higher-level” cognitive processes (facial identity and facial affect recognition). Previous studies using the same cognitive paradigms have indicated that facial affect recognition was most impaired in chronic schizophrenia compared to controls (46) and that disadvantages in facial recognition was a typical feature in children with ASD (47, 48). However, direct comparisons of APS adolescents with and without ASD have not been investigated previously. Based on findings described above, we hypothesized that: 1) facial affect processing would be affected in young adolescents with APS in general, but more strongly related to autism than psychosis on both a categorical and dimensional level; 2) a negative association exists between autistic features and face recognition; and 3) pattern recognition ability would have a stronger, negative impact on facial (affect) recognition in APS without ASD, compared to those with ASD and controls.

## Materials and Methods

### Participants

The study was conducted at the Child and Adolescent Psychiatry Department of the University Medical Center Utrecht. This subsample of the Dutch Prediction of Psychosis Study recruited adolescents (aged 12–18 at intake; M = 15.26, SD = 1.73) putatively at UHR for psychosis. All patient participants were referred help-seekers. Having APS was defined as meeting the Attenuated Positive Symptom Psychosis-Risk Syndrome, as defined in the Criteria of Psychosis-risk Syndromes of the Structured Interview for Prodromal Syndromes (SIPS version 3.0; 49). To fulfil these criteria a patient must receive a rating of level “3”, “4”, or “5” on at least one of the P1-P5 positive symptom items. Having a prepubertal clinical diagnosis of ASD was obtained from information present in the medical records and was confirmed by expert clinical opinion after psychiatric examination including the Autism Diagnostic Interview—Revised (50). The APS groups without and with ASD are from here on referred to as APS/ASD− and APS/ASD+, respectively. Typically developing controls were recruited by distributing information brochures about the research project at several secondary schools in the region of Utrecht. They were excluded if they or a first degree relative had a history of any psychiatric illness, or if they had a second degree relative with a history of a psychotic disorder, as determined by using the Family Interview for Genetic Studies (51). The control group was also screened using the SIPS and individuals were excluded if they met APS criteria. All participants signed an informed consent, and for those younger than 16, the primary caretaker(s) co-signed. The study was approved by the Dutch Central Committee on Research Involving Human Subjects.

### Schizotypal and Autistic Traits

The Schizotypal Personality Questionnaire [SPQ; (52)] is a self-report instrument for assessing trait levels of psychotic-like experiences. It consists of 74 “yes/no” statements addressing the Cognitive-Perceptual Deficits, Interpersonal Deficits, and Disorganization subtraits. This postulated structure of three highly correlated traits has been found to describe clinical data well (53).

The Social Communication Questionnaire (SCQ), previously known as the Autism Screening Questionnaire (54), is a parent-report instrument which addresses autistic traits over the lifetime. Of the 40 “yes/no” questions, all items except 3–8 are scored as reversed. Item 1 was deleted as it is the verbal ability screen for items 2–7, and items 2–7 were deleted for the two cases who responded “no” to item 1. The SCQ has recently been reported to measure three subdimensions of autistic behaviour, namely the Social, Rigidity, and Non-Verbal Communication subtraits, of which the former is moderately correlated with the two latter (55). Like Martin et al. (55), we found items 24 and 25 to have an extremely high tetrachoric sample correlation, but item 25 was retained and assigned to Non-Verbal Communication. Due to the low levels of autistic traits in healthy controls, the parents of that group of participants were not asked to fill in this questionnaire.

### Cognitive Paradigms

Facial affect recognition, face recognition, as well as “lower-level” cognitive skills, namely pattern recognition, were assessed with the Amsterdam Neuropsychological Tasks (ANT), version 2.1 (56). The ANT is a computerized neuropsychological test battery and has proved to be a reliable and valid instrument. Three modules of the ANT were administered for the purpose of this study, namely 1) Feature Identification (FI); 2) Face Recognition (FR); and 3) Identification of Facial Emotions (IFE). The tasks will be described in detail below, for visual representations of the paradigms we refer to Barkhof et al. (46).

The Feature Identification (FI) task consists of 40 trials (20 each of easy and hard conditions) of recognizing a briefly seen target pattern of red and white squares in a subsequently presented 2-by-2 matrix of potential matches. Participants were asked to determine whether the target pattern was present in the 2-by-2 matrix by pressing either YES (target present) or NO (target not present). The target pattern was presented only at the beginning of the task, and had to be kept in mind during the whole task. There were an equal number of target and lure trials, presented in a standard pseudorandom order. Face Recognition (FR) was otherwise similar, but used face stimuli. Participants had to determine whether a target face was present in a set of four, subsequently shown faces. Again, there were 40 trials, half of which required a YES response, and half of which required a NO response, presented in a random order.

Identification of Facial Emotions (IFE) also used the same general setup, but the participant was required to determine whether a target emotion was expressed by a succession of faces that could express any of the following eight emotions: happiness, sadness, anger, fear, disgust, surprise, shame, and contempt. The complete task has eight different parts, i.e., one for each emotion, but only the first four parts were used in the present study, namely happiness, sadness, anger and fear. Each part consisted of 40 trials, half of which were faces that expressed the target emotion (requiring a YES response), and half of which are faces that expressed a random selection of the other seven emotions (requiring a NO response). All trials were analysed jointly for both accuracy as well as speed as a single task.

Individual task results with an error rate of 40% or greater were discarded (one task of one participant in each group), as were those where the task administrator determined that the respondent was confused regarding the instructions (all tasks of one participant in the ASD group). To correct for individual response bias tendencies, the signal detection theory (SDT) discriminability index *d’* was used as the measure of accuracy. To make error-free accuracy possible to score as *d’*, the loglinear transformation rule was applied throughout, as recommended by Hautus (57). In addition to accuracy, response times for correct responses were recorded as a measure of performance. Since response times had skewed distributions, these were converted to response speed (responses per second), which normalized distributions in each group.

Performance in the non-social FI task was used primarily as a baseline for separating the face-specific component of the FR and IFE tasks from its more general visual memory and task performance.

### Statistical Analyses

To assess whether questionnaire responses were sufficiently one-dimensional to use as indicators of single underlying constructs, the included items of the clinical scales were used as categorical indicators of their respective latent variables in separate confirmatory item factor analyses (IFA) using the WLSMV estimator in Mplus 8.3 (58); each item was assigned to a single factor, but all threshold, loading, and factor correlation parameters were freely estimated. Model fit was assessed with the Comparative Fit Index (CFI), the Root Mean Square Error of Approximation (RMSEA), and explained common variance (ECV). Further analyses used the *maximum a posteriori* factor scores derived from these factor analyses. Confirmatory IFA analyses of the published three-dimensional structures were done in a similar manner.

As cognitive task performance was somewhat dependent on age and gender in the control group, all accuracy and speed analyses used standardized regression residuals of *d’* or response speed, that is, the difference between the individual’s observed score and the expected score in the control group for that age and gender. In analyses additionally controlling for FI, the performance on that task was entered as a covariate in the regressions along with age and gender. All analyses additionally controlling for FI use the same data (accuracy/accuracy, and speed/speed).

For group comparisons on the trait and cognitive measures we used *U* tests (as most data was non-normally distributed, and at a conservative *p* < 0.01 due to the number of tests) and reported the corresponding non-parametric effect size *A* (59), which is equivalent to the Area Under the Curve of SDT, and can be estimated with the formula (*n_1_n_2_* − *U*)/*n_1_n_2_*(60). In Cohen’s (61) terminology an effect size *A* = 0.57 (~ d = 0.2) can be considered small, an effect size *A* = 0.64 (~ d = 0.5) can be considered medium and an effect size *A* = 0.71(~ d = 0.8) can be considered large. All analyses except the IFAs were done in IBM SPSS Statistics 26.0. In the primary linear dependence regression analyses, accuracy and response speed were predicted in separate forward-stepping linear regressions by the latent factor scores of the two trait dimensions (SPQ and SCQ). In the similar secondary and exploratory analyses, the predictors were the SPQ and SCQ subdimension factor scores.

## Results

### Subgroup Characteristics

A total of 66 patient participants and 81 healthy controls contributed partial or complete data. Of the patients, 53 had both clinical and cognitive data available, and fulfilled APS criteria, forming the patient subsample for the main analyses. In addition to meeting APS criteria, six patients also met brief, limited, or intermittent psychotic symptoms (BLIPS) criteria as assessed by the SIPS, and three patients also met criteria for genetic risk of psychosis and deterioration (GRD). The patient subsample consisted of adolescents with (n = 21; APS/ASD+ group) and without (n = 32; APS/ASD− group) a diagnosis of ASD. Group characteristics are reported in **Table 1**. APS/ASD− patients were significantly older than APS/ASD+ patients and healthy controls. Both patient groups had somewhat lower IQs than healthy controls. Both APS/ASD− and APS/ASD+ patients showed higher SPQ scores than healthy controls, with no significant difference in SPQ scores between the two patient groups. As expected, the APS/ASD+ group had higher scores on SCQ than the APS/ASD− group.

**Table 1:** Demographic and clinical characteristics with group comparisons.

	Group statistics						Group comparisons (Mann-Whitney *U* test)^††^											
	APS/ASD− (*n* = 32)		APS/ASD+ (*n* = 21)		Controls (*n* = 81)		APS/ASD− vs. APS/ASD+			APS/ASD− vs. Controls					APS/ASD+ vs. Controls			
	% or Mean (SD)		% or Mean (SD)		% or Mean (SD)		*A*	*U*	*p*		*A*	*U*	*p*		*A*	*U*	*p*	
Female	41	%	29	%	51	%	0.56	295.5	0.40		0.55	1166.5	0.41		0.61	663	0.09	
Age	16.4	(1.7)	14.0	(1.7)	15.2	(1.5)	0.83	114	<0.001**		0.69	796	0.001**		0.70	510	0.004*	
IQ	100.3	(14.7)	98.7	(13.3)	107.7	(12.83)	0.51	329	0.90		0.67	861.5	0.005		0.69	530.5	0.01*	
GAF	54.0	(14.3)	58.7	(12.2)	94.0	(7.53)	0.63	249.5	0.12		0.99	35	<0.001**		0.99	21.5	<0.001**	
Current psychotropic medication	37	%	62	%	0	%	0.62	254	0.10		–	–	–		–	–	–	
SPQ score^†^	32.5	(13.8)	30.9	(14.0)	9.3	(7.3)	0.54	297.5	0.68		0.92	196.5	<0.001**		0.94	104	<0.001**	
SCQ score^†^	6.3	(4.8)	16.6	(6.8)	–		0.88	72.5	<0.001**		–	–	–		–	–	–	
SPQ factor score	0.84	(0.64)	0.76	(0.58)	−0.59	(0.70)	0.55	290	0.58		0.94	164	<0.001**		0.95	80	<0.001**	
SCQ factor score	−0.53	(0.67)	0.66	(0.56)	–		0.92	50	<0.001**		–	–	–		–	–	–	
Feature Identification (FI) Discriminability *d’*	3.04	(0.62)	3.22	(0.71)	3.15	(0.59)	0.63	217	0.12		0.56	1106	0.33		0.59	629	0.22	
Feature Identification (FI) Speed**	0.72	(0.11)	0.59	(0.15)	0.71	(0.14)	0.68	191	0.01*		0.53	1190	0.50		0.69	476	0.002*	
Face Recognition (FR) Discriminability *d’*	2.83	(0.61)	2.56	(0.85)	2.89	(0.65)	0.53	298	0.69		0.55	1149	0.40		0.58	665	0.25	
Controlling for FI^†††^	−0.10	(0.63)	−0.23	(0.84)	0.00	(0.64)	0.57	276	0.72		0.57	1104	0.38		0.59	655	0.36	
Face Recognition (FR) Speed**	0.68	(0.16)	0.54	(0.14)	0.72	(0.18)	0.65	221	0.04		0.60	1015	0.07		0.73	432	<0.001**	
Controlling for FI^†††^	−0.05	(0.13)	−0.05	(0.09)	0.00	(0.14)	0.49	325	0.85		0.60	1026	0.09		0.61	627	0.06	
Identify Facial Emotion (IFE) Discriminability *d’*	3.02	(0.59)	2.74	(0.64)	2.90	(0.55)	0.64	231	0.10		0.57	1108	0.23		0.58	684	0.29	
Controlling for FI^†††^	0.18	(0.63)	−0.14	(0.63)	0.00	(0.54)	0.68	202	0.07		0.65	918	0.03		0.57	694	0.51	
Identify Facial Emotion (IFE) Speed**	1.09	(0.18)	0.95	(0.21)	1.10	(0.22)	0.61	249	0.12		0.55	1155	0.37		0.64	577	0.02	
Controlling for FI^†††^	−0.01	(0.15)	0.00	(0.18)	0.00	(0.16)	0.51	316	0.73		0.53	1223	0.65		0.49	832	0.88	

*Significant at p<.01.

**Significant at p<.001.

^†^Estimated with mean substitution.

^††^All statistical tests on cognitive performance used residuals taking into account age and gender (using control group parameters).

^†††^Residuals after taking into account age and gender (using control group parameters), as well as corresponding Feature Identification performance (accuracy/accuracy and speed/speed).

### Data Quality

Age, cognitive variables, and latent psychopathological factors were approximately multivariate normal, and linear regression between them was thus appropriate. The few missing values were treated as being missing at random and all analyses were performed with all available values. The fit of the SPQ and SCQ in unidimensional factor analyses was acceptable (CFI.90/.89, RMSEA.04/.05, with 44%/41% mean explained variance, respectively), and the fit of the three-dimensional models was good (CFI.96/.94, RMSEA.03/.04, with 54%/52% explained variance).

Latent factor scores on the two clinical SPQ and SCQ measures were weakly negatively associated with each other among the patients, Pearson *r* = −0.17 (*p* = 0.25), only the SCQ was predicted by age, *r* = −0. 37 (*p* = 0.01), and there was a trend towards girls having higher SPQ factor scores, *A* = 0.67 (*U* = 204).

### Cognitive Group Comparisons

**Figure 1:**
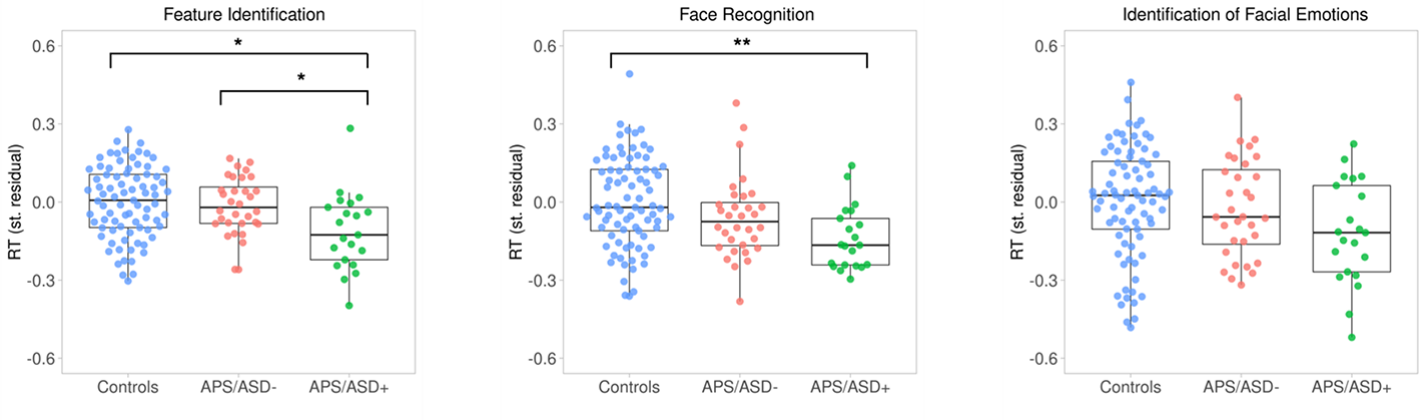
Response times (RT; standardized residuals of responses per second, corrected for sex and age) plotted by group for all three cognitive tasks. Dots represent individual averages with higher scores reflecting faster response times. The summary of the data is shown as a boxplot, with the box indicating the interquartile range (IQR), the whiskers showing the range of values that are within 1.5*IQR and the horizontal line indicating the median. ^*^ *p* <.01; ^**^ *p* <.001.

The cognitive group comparisons are described in **Table 1** and summarized below. Firstly, there was no significant difference between the three groups (APS/ASD− vs. APS/ASD+ vs. Controls) with respect to accuracy on the FI task. However, APS/ASD+ patients were slower in their responses on the FI task than the APS/ASD− and the control groups (**Figure 1**). Secondly, there was no significant difference between the three groups (APS/ASD− vs. APS/ASD+ vs. Controls) in accuracy on the FR task. Again, the APS/ASD+ patients were slower than the other groups (**Figure 1**). However, this difference did not remain significant after controlling for response speed on the “lower-level” FI task. Lastly, no significant group differences were found for the IFE task with respect to either accuracy or response speed. The APS/ASD+ group was again slower in responding than the APS/ASD− group and controls with medium effect sizes, but this difference was not statistically significant.

### Linear Prediction of Social Cognition With Clinical Features

The only cognitive variable which was predicted by SCQ or SPQ factor scores was Feature Identification (FI) accuracy, which was predicted by the SPQ (*β* = 0.37). Secondary analysis revealed that the subfactor Disorganization sufficed to account for this effect (*β* = 0.37).

## Discussion

The aim of this study was to test the hypothesis that facial affect processing is impaired in young adolescents with APS, and whether visual processing at different cognitive levels could help differentiate between those with and without a prepubertal diagnosis of ASD. Neither of the APS groups displayed generalized impairments in the accuracy of facial affect recognition, nor in face or pattern recognition, indicating that these cognitive skills may have limited use as early psychosis vulnerability markers in APS. However, the APS group with ASD generally showed slower responses for affective and non-affective face stimuli than APS participants without ASD and healthy controls, which was fully explained by a slower response time on “lower-level” feature identification.

Contrasting previous findings in UHR samples (62–65), we did not find general emotion processing deficits in the APS groups compared to healthy controls. Given that most individuals with UHR would also qualify for APS, it is unlikely that this stark contrast is due to the inclusion of a more homogenous subset of individuals putatively at-risk. Furthermore, it is undisputed that psychotic conditions are characterized by deficits in facial affect processing (41, 66), but the questions of how and when these deficits manifest remain unresolved so far. A possible explanation of why deviations were not detected in our sample, could be that the number of “false positives” (APS individuals who never convert to psychosis) was too high to be able to discriminate between the APS groups and healthy controls, or perhaps cognitive deterioration only occurs closer to the onset of frank psychosis. Alternatively, facial affect processing difficulties may not emerge until a later age, provided that the involvement of crucial brain structures, such as the amygdala, are still undergoing developmental changes during adolescence (67, 68) that may obfuscate meaningful associations. Finally, due to limited clinical sample sizes, we applied a conservative statistical approach and did not test for emotion-specific variables. There is evidence that facial affect deficits may be more specific in at-risk individuals, e.g. the mislabelling of positive/neutral expressions (37, 40, 69)

Surprisingly, we did not find categorical or dimensional associations with facial emotion or face recognition accuracy in APS with and without ASD. The largest meta-analysis to date directly comparing emotion perception from faces between ASD and SSD subjects reported that both patient groups are impaired and that ASD subjects are significantly more impaired than SSD patients. However, this difference disappears with increasing age (12). The authors suggest that this may reflect a deterioration of social cognitive skills in SSD patients with increasing age, or an age-dependent improvement of emotion perception skills in ASD as a result of social learning. The fact that the APS group without ASD was significantly older in our study could therefore partially explain the negative findings for facial affect recognition. However, we did account for age in our analyses by using standardized regression residuals. Equally striking is the lack of a hypothesized negative relation between face recognition and autistic traits, which has previously been described for this task in ASD populations (47, 48). Together these findings are more in line with the general notion that social cognitive performance in psychosis and ASD are perhaps more similar than dissimilar (15, 70) and teasing this apart may require more refined paradigms, for example by using more ecologically valid stimuli, such as dynamic faces, and by combining them with methods with high temporal resolution, such as EEG or eye-tracking. These commonly available approaches are only just starting to be utilized for direct group comparisons between individuals with ASD and SSD (71–73).

Regarding our third and final hypothesis, no evidence was detected that could indicate basic visual processing may have a stronger, negative impact on facial (affect) recognition in APS without ASD. We did observe a trend towards a significant difference in accuracy, but results were pointing in the opposite direction, i.e. relatively better performance in APS without ASD, and even more so when corrected for pattern recognition. This finding appears to be at odds with common notions of early visual processing difficulties in schizophrenia research (43, 44) and recently also in UHR individuals (39). In contrast to APS individuals without ASD, those with ASD showed slower speed of processing on all cognitive tasks in lieu of typical accuracy. This in line with the notion that it may take more time to process faces in autism, except evidence for this has been inconsistent in ASD (74, 75) and here it appeared to be explained by a more general delay in processing speed, and not by the increased complexity of social stimuli. However, together these findings do suggest that studying the relative impact of psychotic or autistic traits on facial affect processing may benefit from taking into account the individual trade-off between speed and accuracy. Future studies are encouraged to also include a group of ASD individuals without APS to address this issue more thoroughly.

An important limitation of this study is that the use of psychotropic medication was not an exclusion criterion for the psychosis-risk groups, and that the effects of different types of medication on neurocognitive performance are still poorly understood. Secondly, it is possible that the group differences in the present study did not reach statistical significance, because the sample sizes were relatively small. Thirdly, the number of psychotic transitions in this particular APS sample are reportedly low (data available for 10 or less transitions) (27, 76, 77) and could suggest our sample was not highly representative of UHR/APS samples with higher transition rates.

To conclude, this study demonstrated that traditional computerized assessment of facial affect processing is unlikely to detect early vulnerability markers for psychosis in adolescents with APS. A more autistic-like APS profile may be characterized by a generalized increase in response latencies, suggesting that the combined presence of autistic and psychotic traits may disproportionately affect cognitive performance. However, this needs to be replicated with more realistic and dynamic social cognitive stimuli, and supplemented by taking into account speed-accuracy trade-offs. The majority of intervention studies in patients at risk for psychosis focus on a variety of cognitive behavioural therapies for treatment of APS, but until now no specific intervention has been designed for the ASD group (78). Given the elevated risk for psychosis in ASD and our current inability to sufficiently discern between cognitive features in both conditions, there is dire need for more comparative studies to help inform personal treatment guidelines.

## Data Availability Statement

The raw data supporting the conclusions of this article will be made available by the authors without undue reservation.

## Ethics Statement

The studies involving human participants were reviewed and approved by The Dutch Central Committee on Research Involving Human Subjects (CCMO). Written informed consent to participate in this study was provided by the participants’ legal guardian/next of kin.

## Author Contributions 

TZ, HS, and ST planned and designed the study. TZ and HS were involved in subject recruitment and assessment. AM conducted the literature searches. ST undertook the statistical analysis. AM was responsible for drafting the manuscript. All authors contributed to the article and approved the submitted version

## Funding

This work was supported by a project grant from ZON-MW (ZorgOnderzoek Nederland/Netherlands Organization for Scientific Research), project 2630.0001 and by a personal grant for TZ by the Brain & Behavior Research Foundation (NARSAD Young Investigator grant, number 25500).

## Conflict of Interest

The authors declare that the research was conducted in the absence of any commercial or financial relationships that could be construed as a potential conflict of interest.
